# Gel Polymer Electrolytes for Lithium-Ion Batteries Enabled by Photo Crosslinked Polymer Network

**DOI:** 10.3390/gels9120975

**Published:** 2023-12-13

**Authors:** Kyeongsik Kim, Wookil Chae, Jaehyeon Kim, Choongik Kim, Taeshik Earmme

**Affiliations:** 1 Department of Chemical Engineering, Hongik University, Seoul 04066, Republic of Korea; 2 Department of Chemical and Biomolecular Engineering, Sogang University, Seoul 04107, Republic of Korea

**Keywords:** gel polymer electrolytes, lithium-ion batteries, UV polymerization, quasi-solid-state electrolytes

## Abstract

We demonstrate a gel polymer electrolyte (GPE) featuring a crosslinked polymer matrix formed by poly(ethylene glycol) diacrylate (PEGDA) and dipentaerythritol hexaacrylate (DPHA) using the radical photo initiator via ultraviolet (UV) photopolymerization for lithium-ion batteries. The two monomers with acrylate functional groups undergo chemical crosslinking, resulting in a three-dimensional structure capable of absorbing liquid electrolytes to form a gel. The GPE system was strategically designed by varying the ratios between the main polymer backbone (PEGDA) and the crosslinker (DPHA) to achieve an optimal gel polymer electrolyte network. The resulting GPE exhibited enhanced thermal stability compared to conventional liquid electrolytes (LE) and demonstrated high ionic conductivity (1.40 mS/cm) with a high lithium transference number of 0.65. Moreover, the obtained GPE displayed exceptional cycle performance, maintaining a higher capacity retention (85.2%) comparable to the cell with LE (79.3%) after 200 cycles.

## 1. Introduction

Recently, there has been a substantial surge in interest concerning rechargeable batteries, primarily attributed to the substantial rise in the prevalence of mobile devices and, notably, electric vehicles. Lithium-ion batteries (LIBs) stand out as prominent secondary batteries due to their provision of high energy density, light weight, rapid charging capabilities, and versatile applications across a spectrum of domains, ranging from compact electronics to expansive energy storage systems. The burgeoning interest in them, particularly within the automotive sector, is notably driven by endeavors to transition towards electric vehicles (EVs) [1,2].

LIBs conventionally consist of cathodes, anodes, electrolytes, and separators. Electrolytes play a crucial role as mediators facilitating ion transport between cathode and anode. To attain optimal battery performance, it is imperative for electrolytes to exhibit high ionic conductivity (>10^−3^ S/cm) and commendable electrochemical stability. Additionally, they must possess robust thermal stability to ensure battery safety under abnormal conditions. Presently, commercially available LIBs predominantly utilize liquid electrolytes (LEs) due to their advantageous processability with high ionic conductivity. Despite their favorable ionic conductivity, LEs exhibit suboptimal thermal stability at elevated temperatures, consequently giving rise to safety concerns such as leakage, volatility, fire hazards, and explosions, thereby restricting the broader adoption of LIBs across diverse domains [3,4].

In response to these safety challenges, a proposed solution involves the utilization of solid-state electrolytes (SSE) [5,6,7]. SSEs mitigate the risk of leakage inherent in liquid electrolytes, enhancing safety by eliminating susceptibility to external damages. Moreover, SSEs demonstrate heightened thermal stability, thereby averting thermal runaway under extreme conditions. However, typical SSEs, owing to the ion transport constraints within solid particles, manifest relatively low ionic conductivity (~10^−5^–10^−8^ S cm^−1^) compared to conventional liquid electrolytes [8].

Among the various SSEs, solid polymer electrolytes (SPEs) are closed to commercialization due to their processability, excellent foam factor, and good compatibility with electrodes [9]. However, SPEs have a critical problem of low ionic conductivity at room temperature. Much research has been conducted to resolve this issue, such as using filler, modifying the structure, and revealing the mechanism of lithium transport, etc. [10,11,12].

Alternatively, gel polymer electrolytes (GPEs) present a viable option, bridging the properties of liquid and solid electrolytes to effectively address safety concerns and low ionic conductivity [13,14,15]. GPEs permeate a substantial proportion of liquid electrolytes within a polymer matrix, resulting in relatively high ionic conductivity. Furthermore, chemically synthesized polymer matrices of GPEs confer robust properties. The matrix supporting the gel effectively suppresses the growth of lithium dendrites, mitigating the risk of short circuits between electrodes and thereby augmenting battery safety [16,17]. Notably, GPEs offer favorable processability, being producible in film form, rendering them well-suited for large-scale manufacturing [18,19].

**Figure 1:**
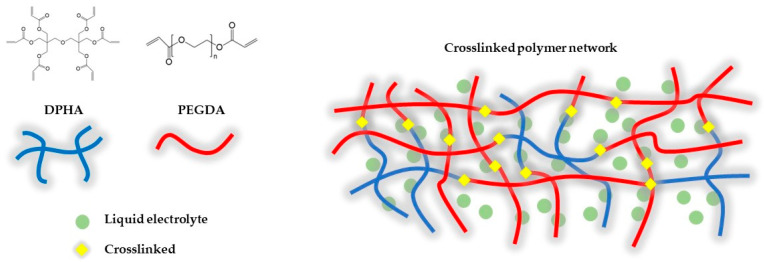
Chemical structure of PEGDA and DPHA with the schematic diagram of the crosslinked polymer matrix network.

Here, we demonstrate GPE with crosslinked polymer matrix by poly(ethylene glycol) diacrylate (PEGDA) and dipentaerythritol hexaacrylate (DPHA) with a radical photo initiator 2-hydroxy-2-methylpropiophenone (HMPP) through ultraviolet (UV) photopolymerization. Figure 1 shows the chemical structures of PEDGA and DPHA and a schematic diagram of the crosslinked polymer matrix network. Two precursors with acrylate functional groups can undergo chemical crosslinking to form a 3D structure that can impregnate liquid electrolytes to form gel. The GPE system was designed by changing ratios between main polymer backbone (PEGDA) and the crosslinker (DPHA) to obtain optimal gel electrolyte. Increased thermal stability compared to conventional LE and high ionic conductivity (1.40 mS cm^−1^) were observed. Furthermore, the obtained GPE showed an excellent cycle performance, maintain higher capacity retention (85.2%) compared to the cell using LE (79.3%) after 200 cycles.

## 2. Results and Discussion

### 2.1. Characterization of GPE

In this study, GPE was subjected to photopolymerization employing ultraviolet (UV) irradiation with wavelength of 365 nm and light intensity approximately 2000 mW cm^−2^. Throughout the polymerization process, the radical species generated by the photo-initiator facilitated the establishment of a three-dimensional (3D) structure via crosslinking of acrylate functional groups within PEGDA and DPHA monomers [20]. The resulting polymer matrix demonstrated the capacity to encapsulate liquid electrolytes, thereby forming a polymeric gel.

**Figure 2:**
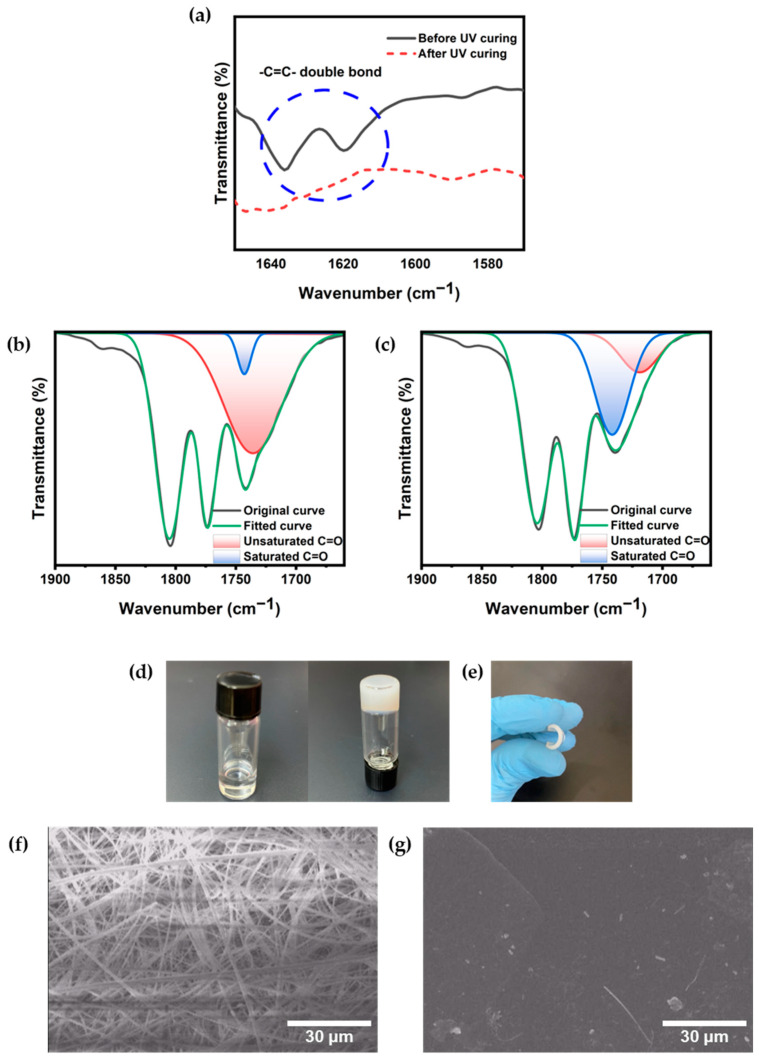
(**a**) FT-IR spectra of precursor solution and formed GPE before and after UV photocuring, respectively; deconvoluted FT-IR spectrum of C=O bond of (**b**) before and (**c**) after polymerization; (**d**) photographs of a precursor solution in a vial before (**left**) and after polymerization (**right**); (**e**) photo of bent GPE film; SEM images of (**f**) pure glass fiber membrane and (**g**) surface of GPE containing glass fiber inside.

The verification of gel electrolyte formation was conducted through Fourier Transform Infrared (FT-IR) spectroscopy, as depicted in Figure 2a. The disappearance of the characteristic double bonds of the acrylate functional group at 1620 and 1640 cm^−1^ served as a discernible indicator of crosslinked gel formation. These peaks, representative of C=C bonds, were evident in the precursor material prior to UV photocuring. Following UV exposure, the two peaks associated with C=C bonds vanished, yielding a smooth spectral curve. The C=O band is also analyzed to observe the change before and after polymerization. To ensure the formation of the polymer matrix, the related area of the unsaturated and the saturated ester C=O bonds are compared. The unsaturated ester C=O bond appears in the range of 1715–1740 cm^−1^, and the saturated ester C=O bond appears in the range of 1735–1755 cm^−1^ [21,22]. To distinguish these overlapped two peaks, the FT-IR spectra in the wavenumber range of 1700 to 1800 cm^−1^ was deconvoluted, as shown in Figure 2b,c. Before polymerization, the area of unsaturated C=O bond was 12.5 times larger than the assumed saturated C=O bond spectrum area (Figure 2b), while the area of the unsaturated C=O bond significantly decreased with the much increased area of saturated C=O bond (Figure 2c). This analytical outcome corroborates the successful photopolymerization process, affirming the generation of a crosslinked polymer matrix.

The liquid precursor solution, comprising liquid electrolyte (LE), PEGDA, DPHA, and photo initiator HMPP exhibited transparency prior to ultraviolet (UV) irradiation. Upon exposure to UV light, the solution underwent a transformative process, resulting in the formation of an opaque GPE with no discernible residual liquid. This GPE exhibited a self-standing and flexible nature, as illustrated in Figure 2d,e. The incorporation of LE into the polymer matrix serves the purpose of mitigating the risk of leakage, thereby reducing potential safety concerns.

Scanning electron microscopy (SEM) images in Figure 2f,g depict the morphologies of a glass fiber membrane and polymerized GPE containing the glass fiber. Notably, GPE polymerized into a smooth and uniform film. One significant challenge encountered in high-energy-density lithium metal electrode-based batteries is the uneven deposition of lithium on the electrode, which leads to dendrite growth. The growth of dendrites can penetrate the separator, causing short circuits. In contrast, the polymer matrix of GPE exhibits a uniform and smooth surface, facilitating the consistent movement of lithium ions, and importantly, its solid-like characteristics characterized by firmness and supportiveness, can physically impede dendrite growth or prevent the separator penetration [23,24]. As a result, Li-ion batteries utilizing GPE are expected to exhibit a higher safety and stability over an extended cycle compared to cells employing liquid electrolytes, which is attributed to these intrinsic advantages.

Within the polymer matrix, the primary monomer is PEGDA, with DPHA serving as a crosslinker by providing a substantial amount of acrylate functional groups. Meanwhile, the photo initiator HMPP facilitates the initiation of the polymerization process. The mechanical properties of the matrix are influenced by the quantity of PEGDA present; an increase in PEGDA content tends to result in a weakening of mechanical properties. Therefore, the incorporation of DPHA, which contributes abundant acrylate functional groups, acts to complement, and enhance the mechanical properties of the matrix through more intense crosslinked structures of the 3D matrix.

It is noteworthy that an elevation in the quantity of DPHA leads to the formation of a rigid matrix, rendering it more brittle and susceptible to fracture. Consequently, careful consideration of the PEGDA to DPHA ratio is crucial to achieve a balanced matrix. In this study, three specific ratios, namely GPE 3:1, 4:1, and 5:1, were selected to ensure the self-standing gel films with sufficient flexibility that would not break when bent 180 degrees, and residual electrolytes did not remained on the surface of the film after gelation. The GPEs were further analyzed to determine the optimal composition for achieving suitable mechanical properties as well as cell performance.

**Figure 3:**
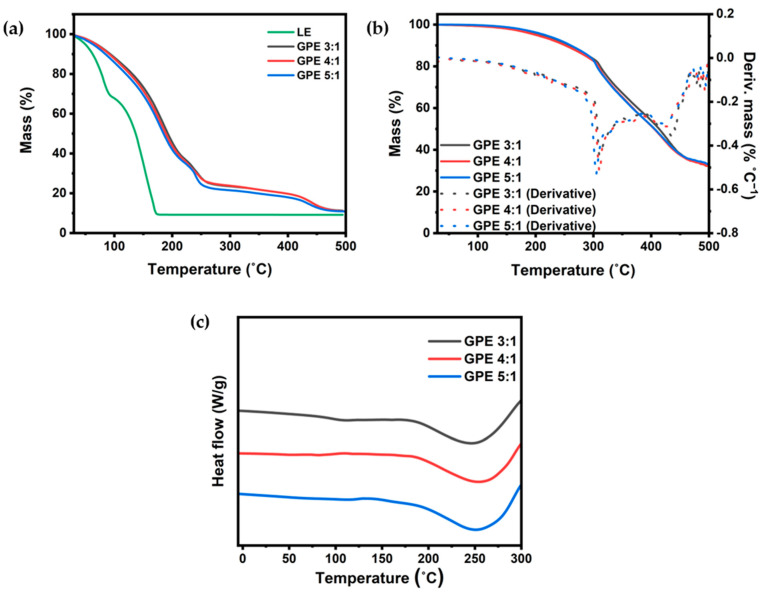
(**a**) TGA curves of liquid electrolyte and GPEs with different monomer ratios; (**b**) TGA curves of polymer matrices without liquid electrolytes; (**c**) DSC curves of pure GPEs with different ratios.

Thermogravimetric analysis (TGA) was conducted to compare the thermal stability of GPE with LE. Observing changes in mass with temperature enables the assessment of the thermal stability of the substance by tracking evaporation and decomposition temperatures. As shown in Figure 3a, the LE experienced decomposition starting around 50 °C, while GPEs initiate decomposition at temperatures exceeding 65 °C. The LE curve exhibits a significant decomposition before reaching 100 °C, whereas the GPEs extend beyond 200 °C. Notably, the overall mass reduction pattern of GPE has shifted to higher temperatures. The disparity in TGA curves between LE and GPEs is likely attributed to the presence of a polymer matrix, which can delay the evaporation and decomposition of LE. Through the formation of a polymer matrix with solid-like properties via crosslinking, an enhancement in the thermal stability of GPE has been observed. The thermal stability of the polymer matrix without liquid electrolyte was also assessed. The polymer matrix was obtained by washing GPE, followed by drying. Figure 3b illustrates the TGA results of the polymer matrix at various ratios. It is evident from the results that the mass loss observed between 110 °C and 300 °C for ratios of 3:1, 4:1, and 5:1 is presumably attributed to the decomposition of LiPF_6_ (decomposition temperature: 106 °C) and residual solvent present in the matrix. The polymer matrix undergoes decomposition at 300 °C, as indicated by the peak in the first derivative of the mass.

The polymer matrix, exhibiting a high thermal decomposition temperature of 300 °C, holds promise for the development of a thermally stable GPE. Enhancing the stability of GPE under high-temperature conditions can be achieved by optimizing the solvent used in the preparation of the polymer electrolyte or by selecting a thermally superior solvent.

To analyze the characteristics of the polymer matrix of GPEs, we conducted differential scanning calorimetry (DSC) analysis to investigate the influence of the PEGDA to DPHA ratio. The crystallinity of the polymer matrix can be analyzed using melting temperature in DSC. Through the shift of melting temperature, the increase and decrease in the amorphous region of the polymer matrix can be identified, and the increase in the amorphous region could cause the decrease in melting temperature [25,26]. Figure 3c illustrates that the GPEs exhibit a melting temperature around 250 °C. A slight discrepancy in melting points is observed, with GPE 3:1 below 250 °C, while GPE 4:1 and 5:1 surpass this threshold. The higher melting point correlates with increased crystallinity, suggesting that the addition of PEGDA results in enhanced crystallinity. Like the previous report of the polyethylene oxide (PEO) groups in PEGDA increasing, crystallinity tends to rise, while the addition of DPHA can decrease crystallinity [27]. The results show that altering the PEGDA:DPHA ratio modifies the number of acrylate functional groups, affecting the density of the matrix and thus influencing the characteristics of GPE.

### 2.2. Electrochemical Properties of GPEs

**Figure 4:**
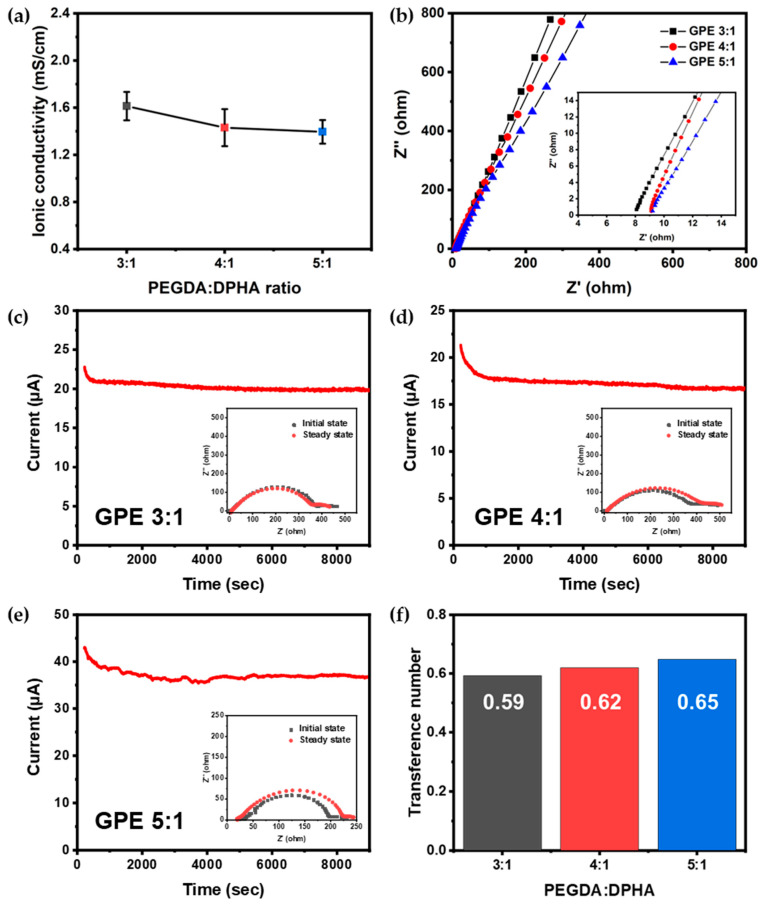
Electrochemical properties of GPEs: (**a**) ionic conductivities of GPEs; (**b**) Nyquist plot of GPEs; chronoamperometry and impedance measurement of symmetric Li|GPE|Li cells with (**c**) GPE 3:1, (**d**) GPE 4:1, and (**e**) GPE 5:1; (**f**) Li^+^ transference numbers of GPEs at various ratios.

Ionic conductivity stands as a crucial parameter in LIBs, given the pivotal role of electrolytes in facilitating the rapid transport of ions between electrodes. Typically, LEs exhibit ionic conductivity within the range of 10^−3^–10^−2^ S cm^−1^, whereas GPEs with ionic conductivity surpassing 10^−3^ S cm^−1^ are considered sufficiently high. Ionic conductivities were measured using a stainless steel (SS)|GPE|SS cell via AC impedance spectroscopy with varying PEGDA and DPHA ratios. Figure 4a illustrates the measured ionic conductivities, while Figure 4b presents the Nyquist plot of GPE.

The average ionic conductivities of GPE 3:1, 4:1, and 5:1 were 1.61, 1.43, and 1.40 mS cm^−1^, respectively. All GPE compositions exhibit high ionic conductivity exceeding 10^−3^ S cm^−1^ with an 85% composition of LE in the GPE, with the average thickness of the polymerized GPEs with glass fibers inside being 0.435 (±0.022) mm. According to the DSC results, GPE 3:1 demonstrates rather low crystallinity, resulting in relatively low bulk resistance and consequently achieving high ionic conductivity. For GPE 4:1 and 5:1, the crystallinity of the polymer matrix is very similar, leading to comparable ionic conductivity. These results demonstrate an ionic conductivity of GPEs more than 100 times higher than that of solid electrolytes.

The Li^+^ transference number (t^+^) signifies the ratio of mobility of lithium cations within the electrolyte containing dissolved lithium salt. We constructed a Li metal symmetrical cell and measured the Li^+^ transference number. As illustrated in Figure 4c–f, the matrices composing GPEs at ratios of 3:1, 4:1, and 5:1 exhibited high Li^+^ transference numbers of 0.59, 0.62, and 0.65, respectively, all over 0.5. Acrylate functional groups containing C=O group in PEGDA and DPHA facilitate the solvation of lithium ions from LiPF_6_. Moreover, the oxygen atoms in the repeating poly(ethylene oxide) backbones of PEGDA during the ion transfer process are presumed to assist the movement of lithium cations, contributing to a high Li^+^ transference number. Furthermore, when a polymer matrix is formed, the movement of anions, which are relatively large compared to lithium cations, can be hindered, potentially contributing to the high Li^+^ transference number in GPE [28,29,30].

An augmentation in the quantity of PEGDA correlates with an enhanced Li^+^ transference number. As mentioned above, a higher PEGDA content is linked with increased crystallinity. This indicates a heightened degree of crosslinking due to acrylate functional groups, leading to the formation of a denser and more compact polymer matrix. Under these conditions, the movement of anions can be more restricted, while cations can move more freely. Therefore, a higher PEGDA content is speculated to result in a higher Li^+^ transference number. Comparatively, the measured Li^+^ transference number of GPE 5:1 stands on the higher side when compared to reported Li^+^ transference numbers in previously studied gel electrolytes, especially in contrast to electrolytes based solely on PEGDA. It is at a similar level to electrolytes that enhance transport by incorporating additional materials in the polymeric electrolytes [14,31,32].

The characteristics of the GPEs are summarized Table 1, demonstrating the GPEs to be electrolytes with high ionic conductivity and Li^+^ transference numbers.

### 2.3. Battery Cell Performance Using GPE as an Electrolyte

**Figure 5:**
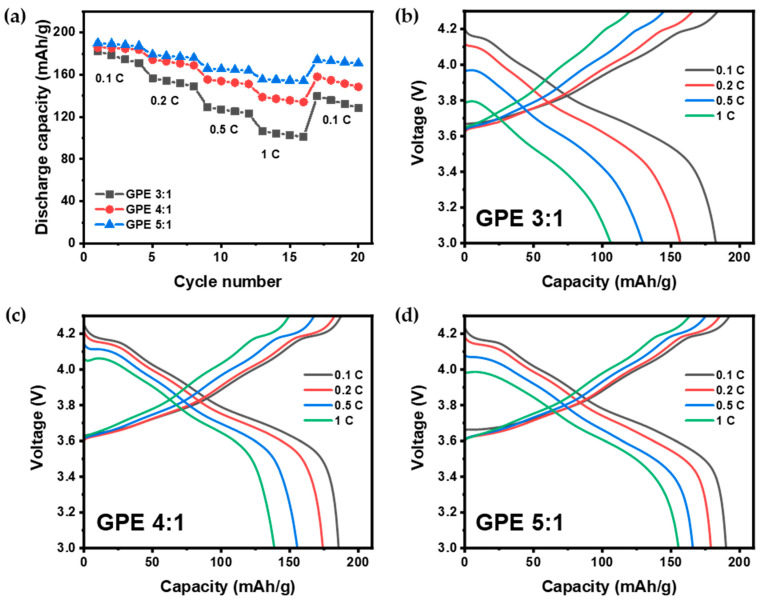
Charge–discharge characteristics of the NCM811|GPE|Li cells at different rates: (**a**) discharge capacities of GPEs at different ratios; charge–discharge voltage profiles of (**b**) GPE 3:1, (**c**) GPE 4:1, and (**d**) GPE 5:1.

We fabricated an NCM811|GPE|Li coin cell and conducted a rate capability test, measuring the capacity with different ratios while varying discharge rates. The results are presented in Figure 5a. The GPE 3:1 ratio exhibited capacities of 182.6, 156.8, 129.4, and 106.2 mAh g^−1^ at discharge rates of 0.1, 0.2, 0.5, and 1 C-rate, respectively (Figure 5b). GPE 4:1 showed capacities of 185.6, 174.1, 155.5, and 138.8 mAh g^−1^ at the same discharge rates (Figure 5c), while GPE 5:1 demonstrated capacities of 190.0, 179.0, 165.8, and 155.5 mAh g^−1^ at 0.1, 0.2, 0.5, and 1 C discharge rates, respectively (Figure 5d).

The capacity retention at a high discharge rate of 1 C-rate, relative to 0.1 C-rate, was found to be 58.2%, 74.8%, and 81.8% for GPE 3:1, 4:1, and 5:1, respectively. Notably, GPE 5:1 exhibited the highest capacity retention even at high rates. Furthermore, after completing the test at a high discharge rate and discharging again at 0.1 C-rate, GPE 5:1 demonstrated the most robust recovery, with capacities of 174.0 mAh g^−1^, achieving a recovery of 91.6%, compared to 139.7 mAh g^−1^ (76.5% recovery) for GPE 3:1 and 158.1 mAh g^−1^ (85.2% recovery) for GPE 4:1. GPE 5:1 demonstrated stable capacity even at high rates in the rate capability test, presenting excellent recovery. The results are consistent with the higher Li^+^ transference number of GPE 5:1. The resistances of the GPE 5:1 battery was 306.2 Ohm before initial charging and 393.8 Ohm after 50 cycles.

**Figure 6:**
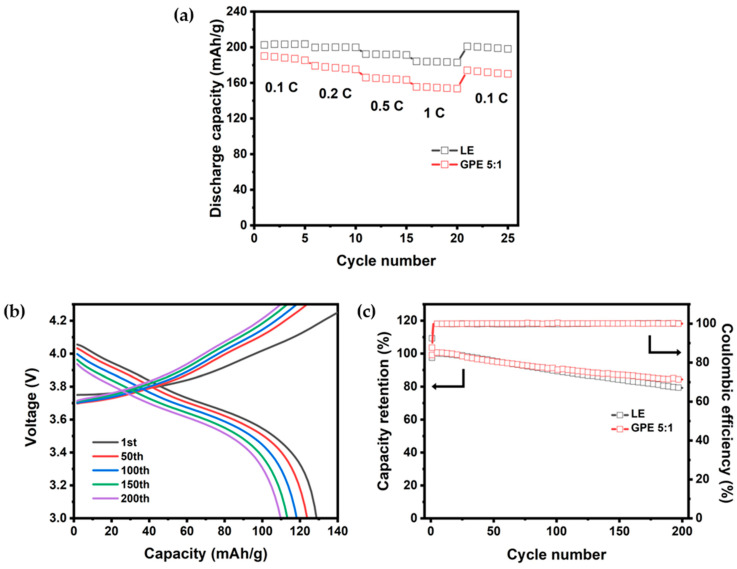
Electrochemical performance of NCM811|LE|Li and NCM811|GPE|Li cells: (**a**) rate capability of cells using LE and GPE 5:1; (**b**) charge/discharge voltage profile of cell using GPE 5:1 with cycle number at 0.5 C-rate; (**c**) cycle characteristics of cells using LE and GPE 5:1 at 0.5 C-rate.

Subsequently, the battery performance of GPE 5:1 was compared with a cell using a liquid electrolyte (LE). The results of this comparison are presented in Figure 6a. At a 0.1 C-rate, the discharge capacities for the LE and GPE were 202.5 and 190.0 mAh g^−1^, respectively. At a 1 C-rate, the discharge capacities were 184.0 mAh g^−1^ for the liquid electrolyte and 155.5 mAh g^−1^ for GPE. The discharge capacity values indicate that the cells using a liquid electrolyte maintained a larger capacity. LEs can sustain high capacities owing to their high ionic conductivity and liquid state.

However, the cell with GPE 5:1 also exhibited a respectable capacity and demonstrated stable capacity retention even at high discharge rates without drastic changes. Comparing the discharge capacities at 0.1 C-rate to 1 C, it was observed that the capacity decrease was relatively similar for both LE and GPE cells. The 0.5 C/0.5 C charge/discharge voltage profile of the coin cell composed of NCM 811|GPE|Li is shown in Figure 6b. The initial discharge capacity was 128.8 mAh g^−1^, 123.8 mAh g^−1^ (retention ratio: 96.1%) at 50th, 118. 2 mAh/g (retention ratio: 91.8%) at 100th, 113.3 mAh g^−1^ (retention: 88.0%) at the 150th, and 109.7 mAh g^−1^ (retention ratio: 85.2%) at 200th cycle, with a stable voltage profile from 1 to 200 cycles. Additionally, an NCM811|GPE|Li coin cell was charged/discharged at a 0.5 C-rate to assess cycle performance, comparing its discharge capacity retention with that of a cell using liquid electrolyte. The results are presented in Figure 6c. After 100 cycles, both LE and GPE cells exhibited 89.6% and 91.8% capacity retention, respectively, relative to their initial capacities. After 200 cycles, these values were 79.3% and 85.2%, respectively. Furthermore, both cells demonstrated an average Coulombic efficiency of 99.9% throughout the cycle tests. The cell with GPE demonstrated excellent cycle performance comparable or even surpassing that of liquid electrolyte systems. This represents a stable transportation of lithium ions within the liquid electrolyte contained within the matrix of the GPE during charge/discharge processes through cycle performance evaluations. Furthermore, prior investigations into GPE systems utilizing NCM811 as a cathode have indicated that capacity retention typically did not exceed 80% after 200 cycles [33,34,35]. Nevertheless, the GPE investigated in this study demonstrated outstanding cycle performance over 200 charge and discharge cycles, achieving an 85% capacity retention. This accomplishment represents a significant advancement in battery performance and reliability, marking a crucial step forward for future research aiming to enhance battery technologies.

## 3. Conclusions

In this study, we have successfully demonstrated a gel polymer electrolyte (GPE) with a crosslinked polymer matrix through UV photopolymerization. Through thermal analysis (TGA and DSC), we have confirmed the better thermal stability of the GPE compared to the liquid electrolyte, attributing this to the supportive nature of the polymer matrix that integrates and reinforces the liquid electrolyte. The GPE exhibited high ionic conductivity at 25 °C (1.40 mS cm^−1^), rendering it suitable for Li-ion battery applications. Additionally, it demonstrated a high Li^+^ transference number (t^+^ = 0.65) despite the quasi-solid-state phase of the GPE. The fabricated NCM811|Li metal half coin cell utilizing the GPE showed stable discharge capacity, even at high discharge rates, comparable to that of the liquid electrolyte cell. Furthermore, during the long-term cycling performance evaluation at a 0.5 C-rate, the GPE-based cell exhibited impressive capacity retention, reaching 85% of its initial discharge capacity. The demonstrated GPE shows a great potential as a secure and reliable replacement for conventional liquid electrolytes in future battery applications.

## 4. Materials and Methods

Poly(ethylene glycol) diacrylate(PEGDA) M_n_ = 250, 2-Hydroxy-2-methylpropiophenone (HMPP), and ethylene carbonate (EC):diethyl carbonate (DEC) = 1:1 with 1 M LiPF_6_ electrolyte were purchased from Merck KGaA, Karmstadt, Germany. Dipentaerythritol hexaacrylate (DPHA) was purchased from Tokyo Chemical Industry (TCI), Tokyo, Japan. All chemicals were used without any further purifications. GPE precursor solution was prepared by mixing liquid electrolyte, PEGDA, DPHA, and HMPP and stirred for 2 h at room temperature. The composition of the liquid electrolyte in the precursor was 85 wt%, and the polymer matrix with PEGDA and DPHA constituted 15 wt%, with 1 wt% HMPP within the polymer matrix. GPE 3:1, 4:1, and 5:1 indicate PEGDA to DPHA ratios of 3:1, 4:1, and 5:1 by weight ratios.

For preparation of slurry for cathodes, NCM811, PVDF, and carbon black (all purchased from Wellcos Co., Gunpo-si, Republic of Korea) were mixed 8:1:1 by weight ratio in *N*-methyl-2-pyrrolidone (NMP) solvent (Merck KGaA, Karmstadt, Germany). Then, the prepared slurry was coated on a cleaned aluminum foil using a doctor blade and dried for 12 h in a vacuum oven at 100 °C. To fabricate coin cells, a NCM811 cathode was positioned within a coin cell case and covered with glass fiber. Subsequently, the precursor solution was injected, and UV light was illuminated for 40 s using UV LED (Lichtzen, INNOCURE HQ-100, Gunpo-si, Republic of Korea). The Li metal anode was then placed on the polymerized GPE, and the coin cell was assembled inside Ar gas filled glovebox. For the cells using LE, liquid electrolyte was injected into the cell instead of GPE precursor solution without UV illumination.

The morphologies of the GPE samples were observed using a scanning electron microscopy (SEM, Hitachi, S-4500, Tokyo, Japan). The FT-IR analysis was conducted from 500 to 4000 cm^−1^ (Perkin Elmer, Spectrum 3, Seoul, Republic of Korea). Thermal properties were measured using TGA (Netzsch, TGA 209) from 30 to 500 °C at the rate of 20 °C/min and DSC (TA instruments, DSC 25, New Castle, DE, USA) from −90 to 350 °C at the rate of 10 °C/min under N_2_ atmosphere. Ionic conductivities of GPE were measured using AC impedance spectroscopy (Yong In AT Co. Ltd., AMETEK VersaSTAT 3, Anyang-si, Republic of Korea) with SS|GPE|SS stainless steel sandwiched cell and calculated by Equation (1),

(1) $$\sigma =\frac{1}{R_{b}}\times \frac{l}{A}$$

where *l* is the thickness of the electrolyte, *A* is the area of the stainless steel, *R_b_* is the bulk resistance of the electrolyte. The Li^+^ transference number was calculated by the Bruce–Vincent–Evans Equation (2),

(2) $$t_{Li+}=\frac{I_{ss}(∆V-I_{0}R_{0})}{I_{0}(∆V-I_{SS}R_{SS})}$$

where ∆*V* is potential applied (10 mV); *I*_0_ and *I_SS_* are the initial and steady state current, respectively; and *R*_0_ and *R_SS_* are the interfacial resistance before and after the polarization.

For every assembled coin cell, a galvanostatic charge–discharge test was conducted with a battery test system (WBCS3000Le, WonATech, Seoul, Republic of Korea) in the voltage range from 3.0 to 4.3 V, initially charged/discharged at a 0.1 C-rate for 2 cycles. Then, the coin cells were charged at a 0.1 C-rate and discharged at different C-rates of 0.1, 0.2, 0.5, and 1 C-rate for the rate capability test. To evaluate cycle performance, the coin cells were charged/discharged at a 0.5 C-rate.

## Figures and Tables

**Table 1:** Summary of GPE characteristics.

Sample	Decomposition Temperature ^1^	Melting Temperature	Ionic Conductivity	Li^+^ Transference Number
GPE 3:1	69.6 °C	246 °C	1.61 (±0.12) mS cm^−1^	0.59
GPE 4:1	69.0 °C	251 °C	1.43 (±0.16) mS cm^−1^	0.62
GPE 5:1	67.0 °C	252 °C	1.40 (±0.1) mS cm^−1^	0.65

^1^ The temperature when the sample has lost 5% of initial weight.

## Data Availability

The data presented in this study are available on request from the corresponding authors. The data are not publicly available due to ongoing research using a part of the data.
